# Hybrid Composites Based on Polypropylene with Basalt/Hazelnut Shell Fillers: The Influence of Temperature, Thermal Aging, and Water Absorption on Mechanical Properties

**DOI:** 10.3390/polym12010018

**Published:** 2019-12-20

**Authors:** Anna Kufel, Stanisław Kuciel

**Affiliations:** Faculty of Materials Engineering and Physics, Institute of Materials Engineering, Tadeusz Kosciuszko Cracow University of Technology, Al. Jana Pawła II 37, 31-864 Cracow, Poland; stask@mech.pk.edu.pl

**Keywords:** hazelnut shell, basalt fiber, polypropylene, hybrid composites, natural filler, thermoplastic

## Abstract

The aim of the research was to study the effects of adding natural fillers to a polypropylene (PP) matrix on mechanical and physical properties of hybrid composites. The 10%, 15%, and 20% by weight basalt fibers (BF) and ground hazelnut shells (HS) were added to the PP matrix. Composites were produced by making use of an injection molding method. Tensile strength, tensile modulus, strain at break, Charpy impact strength, and the coefficient of thermal expansion were determined. The influence of temperature, thermal aging, and water absorption on mechanical properties was also investigated. In addition, short-time creep tests were carried out. To characterize the morphology and the filler distribution within the matrix, a scanning electron microscope (SEM) was used. The results showed that the addition of the two types of filler enhanced mechanical properties. Furthermore, improvements in thermal stability were monitored. After water absorption, the changes in the tensile properties of the tested composites were moderate. However, thermal aging caused a decrease in tensile strength and tensile modulus.

## 1. Introduction

In recent years, natural fibers have increasingly been used as an alternative to traditional synthetic fibers, which have harmful effects on the environment. Furthermore, agricultural waste is used to manufacture composites due to an increasing demand for eco-friendly materials. Composites may be reinforced (e.g., by wheat husk and rye husk [1], rice husk [2], cocoa pod husk [3], or spent coffee ground [4]). Walnut shells are renewable lignocellulosic materials and they can be obtained as agricultural by-products. In 2017, the world production of hazelnuts (in shells) was 1 million tonnes [5]. Hazelnut shells (HSs) are characterized by low density; as reported by Copur [6], the density of hazelnut shells is 0.23 g/cm^3^ (±0.21), which appears to be a lower value compared to raw wood material (0.40–0.75 g/cm^3^). Gurbanow et al. [7] studied polypropylene (PP) composites with hazelnut shell flour. It was found that HSs can be used as a filler in polymer composites. In another study [8], HSs were used in bio-based composites based on polylactic acid. The addition of HSs increased the stiffness of the composites. Among various natural fibers, basalt fibers (BFs) have been extensively used in various fields of engineering. BFs are mineral fibers with mechanical properties, similar to glass fibers, and low-cost compared to carbon fibers [9]. BFs have good physical, chemical, and thermal properties as well as excellent mechanical and chemical resistance. Basalt is a natural material, and no chemical additives, solvents, pigments, or other hazardous materials, are used in the production of its fibers. Caused by that fact, BF can be considered a sustainable material [10]. Composites reinforced with BFs are widely used due to their high mechanical properties and thermal stability; additionally, they are eco-friendly materials. Many researchers have studied composites reinforced by BFs [11,12,13]. Manikandan et al. [14] investigated the effect of surface modifications of BFs on mechanical properties. The results showed that acid treatment of BFs improved tensile strength and impact strength. Yang et al. [15] added carbon nanotubes and aluminum hypophosphite to polylactide/BF composites, which reduced the peak heat release rate of the composite. Hybrid composites offer better balance in mechanical properties than non-hybrid composites. Predicting their mechanical properties is challenging due to the synergistic effects between both fibers [16]. Hybridization provides us with the opportunity to improve some of the properties of composites. It is also common to reduce production costs by replacing the more expensive filler with a cheaper filler. By using hybridizations, composites can be produced that combine the features of both fillers. An addition of carbon fibers with smaller diameters and larger and longer BFs to polyamide 6, improved mechanical properties of composites [17]. Mechanical properties of natural filler composites are lower compared to synthetic fibers. Furthermore, natural fillers absorb moisture. The amalgamation of two different types of fillers in the same matrix can result in a material that overcomes these drawbacks. A few studies reported hybrid composites reinforced by both natural and synthetic fillers. Samal et al. [18] studied hybrid PP composites with banana and glass fibers. In another study [19], mechanical properties and the water absorption of high-density polyethylene composites, reinforced by wood and carbon fibers, were investigated. An addition of a small amount of carbon fibers to wood plastic composites enhanced the mechanical properties without additional operating costs. In another study [20], effects on mechanical performance of composites with man-made cellulose and glass fibers were investigated.

There is research on composites reinforced with HSs and BFs; however, hybrid composites with these fillers have not been studied so far. This study aimed to investigate the influence of an addition of natural fillers to a polypropylene matrix on mechanical properties. Furthermore, the effect of temperature, water absorption, and thermal aging was studied. Additionally, such properties as thermal conductivity, thermal expansion, and creep behavior were monitored. The characterization of an adhesion of fillers with the matrix by scanning electron microscope (SEM) was observed.

## 2. Materials and Methods

### 2.1. Materials

PP Moplen HP500N produced by Basell Orlen Polyolefins (Płock, Poland) was used as a matrix. The following fillers and coupling agents were used:

1. The chopped basalt fibers (BCS17-6.4-KV16) with a cutting length of 6.4 mm and a nominal diameter of 17 μm were supplied by Basaltex (Wevelgem, Belgium).
2. Hazelnut shells were ground on a Retsch EMAX mill (Retsch GmbH, Haan, Germany). The particle size was from 90 to 200 μm. The shells were treated with 5% NaOH for 1 h, rinsed with water, and dried for 2 h at 90 °C in a molecular drier.
3. The maleic anhydride PP (MAPP) PP SCONA TPPP 9112 GA was supplied by Byk (Altana AG, Wesel, Germany).

### 2.2. Methods of Testing

#### 2.2.1. Preparation of Composite Specimens

Composites were prepared in the Laboratory of Plastics Technology operating under Grupa Azoty S.A. in Tarnów, Poland. The BFs, HSs, and PP were compounded on a MARIS TM30 co-rotating twin-screw extruder (Maris America Corp., Windsor Mill, MD, USA). The samples were made by using an Engel ES 200/40 HSL injection-molding machine (ENGEL GmbH, Schwertberg, Austria). The following parameters were set: temperature from 180 to 220 °C, injection speed from 60 to 90 mm/s, rotation speed of the screw of 63 rpm, a cycle time of 60 s, and a mold temperature of 40 °C.

#### 2.2.2. Density

The density (PN-EN ISO 1183) of the samples was estimated using the hydrostatic method on a RADWAG WAS 22W scale (Radwag, Radom, Poland). The specimens were measured in an ethanol medium.

#### 2.2.3. Vicat Softening Temperature Measurement

The softening temperature was tested with a Ceast Heat Deflection Temperature (HDT) and Vicat Tester Type 6520 (CEAST, Pianezza, Italy) machine. The measurements were done using method A50, according to PN-EN ISO 306:2006. The 10 N was applied. The bath temperature was raised to 50 °C/h until the needle penetrated 1 mm.

#### 2.2.4. Mechanical Testing

The mechanical properties were analyzed using an MTS Criterion Model 43 universal testing machine (MTS Systems Corp., Eden Prairie, MN, USA) with an MTS 634.31F axial extensometer, according to PN-EN ISO 527-1:2012. The constant cross-head speed was set to 5 mm/min. Charpy impact tests (PN-EN ISO 179-2) were carried out on unnotched samples using the Zwick/Roell MTS SP (Zwick Roell Group, Ulm, Germany) testing machine. Furthermore, the tests were performed after 30 days of water absorption, 144 h of aging, and at various temperatures (−24, 23, and 80 °C).

#### 2.2.5. Thermal Conductivity

The thermal conductivity was measured with the hot disk thermal constants analyzer transient plane source (TPS) 500 (Hot Disk AB, Göteborg, Sweden) in accordance with ISO 22007-2.

#### 2.2.6. Thermal Expansion

The analysis of composites was carried out on the NETZSCH 402 F1 Hyperion device (NERZSCH Group, Selb, Germany). The samples were placed vertically. The test parameters were recorded with Proteus software. The test temperature was between −60 and 140 °C. The heating and cooling was set at 10 °C/min. The coefficient of linear thermal expansion (LTCE) was computed using the following formula:

(1) $$\propto_{L}=\frac{1}{L}\frac{dL}{dT},\;1/K$$

where *L* is the linear dimension of the specimen and d*L*/d*T* is the rate at which the linear dimension changes per unit of temperature.

#### 2.2.7. Creep Test

The short-time creep tests were performed using an electromechanical Zwick 2.5 testing machine (Zwick Roell Group, Ulm, Germany), according to PN-EN ISO 899-1:2005. Creep experiments at 23 °C were carried out under an applied stress of 15 MPa.

#### 2.2.8. Composite Morphology

The microstructure observations were made with the use of a scanning electron microscope JEOL JSN5510LV (JEOL Ltd., Tokyo, Japan). SEM structure images were acquired on tensile-test fracture surfaces of specimens. The accelerating voltage was 20 kV. Samples were coated with gold using a Cressington 108 auto sputter coater (Cressington Scientific Instruments, Watford, UK).

#### 2.2.9. Water Absorption

Water absorption, after soaking in distilled water for 1, 7, 20, and 30 days, was carried out according to the ASTM D570-98 standard. An electronic weighing balance (RADWAG WAS 22W) was used. The following equation was used to calculate the water absorption (%*W*):

(2) $$\%W=\frac{W_{n}-W_{0}}{W_{0}}\times 100$$

where *W*_0_ and *W*_n_ is the weight of the sample before and after soaking in water, respectively; %*W* is the percentage increase in weight.

#### 2.2.10. Thermal Aging

**Figure 1:**
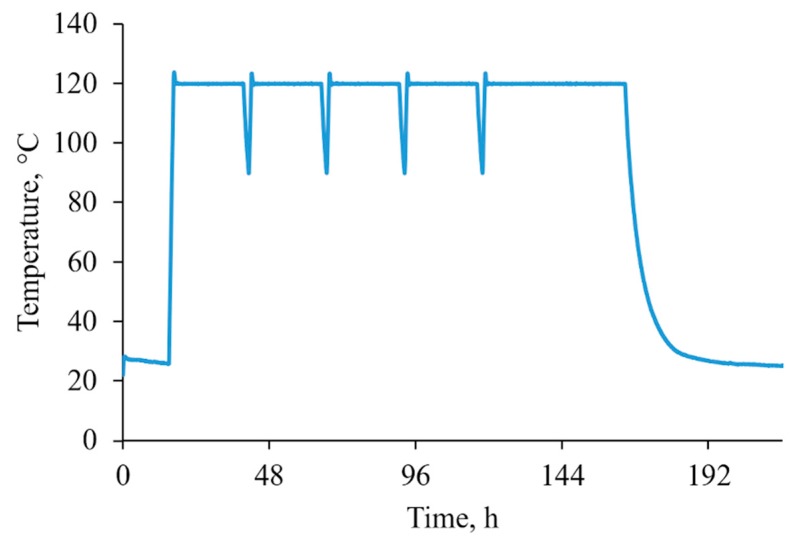
Diagram of the accelerated aging process in an autoclave.

**Figure 2:**
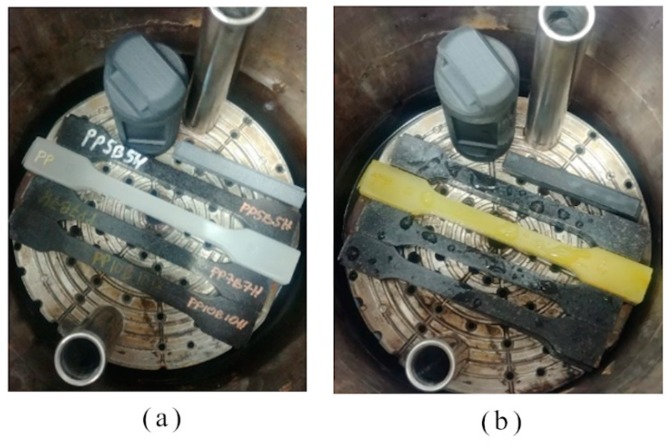
The appearance and arrangement of samples in an autoclave (**a**) before and (**b**) after the accelerated aging process.

Accelerated aging tests were realized using an autoclave (Parr Instrument Company, Moline, IL, USA) according to the EN ISO 2440 standard. Samples were conditioned for 16 h in the temperature of 23 ± 2 °C and a humidity that was equal to 50 ± 5%. Temperature was set at 120 °C, the humidity was 100%, and the pressure was 0.3 MPa. The aging process lasted for 144 h. A diagram of the accelerated aging process is shown in Figure 1. The arrangement of the samples in the autoclave is shown in Figure 2.

## 3. Results and Discussion

### 3.1. Physical and Processing Properties

Three different ratios were used to produce composites. A detailed description of the composition with the density measurement and Vicat softening temperature is given in Table 1. The addition of fibers caused a slight increase in the density of composites. The density of composites containing 20 wt% fillers was 12.5% higher than for unfilled PP. However, hybrid composites with BFs and HS particles are still lightweight materials. The softening point was also determined. The Vicat softening temperature increased up to 161.8 °C for the PP10B10H composite.

### 3.2. Mechanical Properties

**Figure 3:**
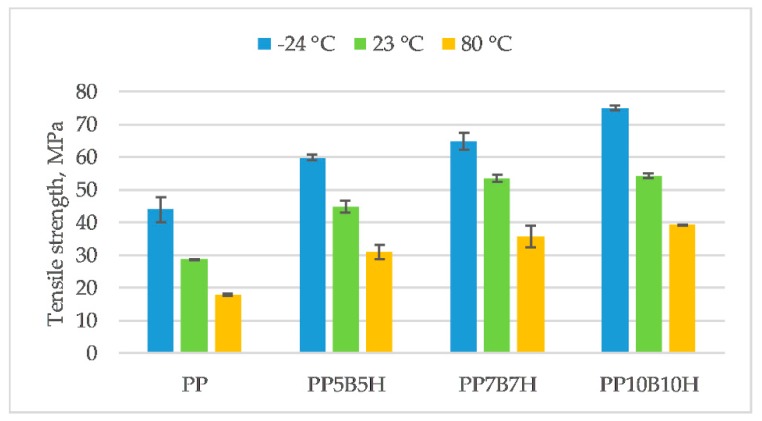
Tensile strength of the tested materials at −24, 23, and 80 °C.

**Figure 4:**
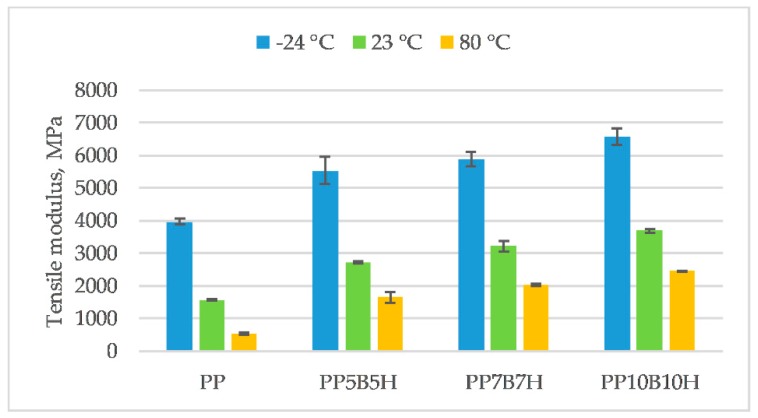
Tensile modulus of the tested materials at −24, 23, and 80 °C.

**Figure 5:**
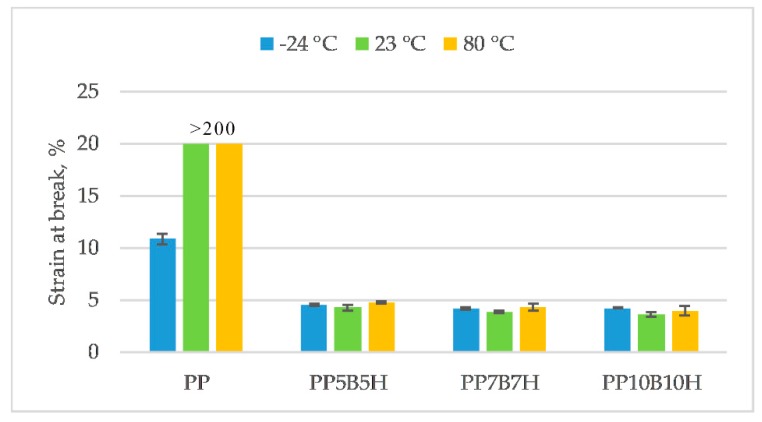
Strain at break of the tested materials at −24, 23, and 80 °C.

**Figure 6:**
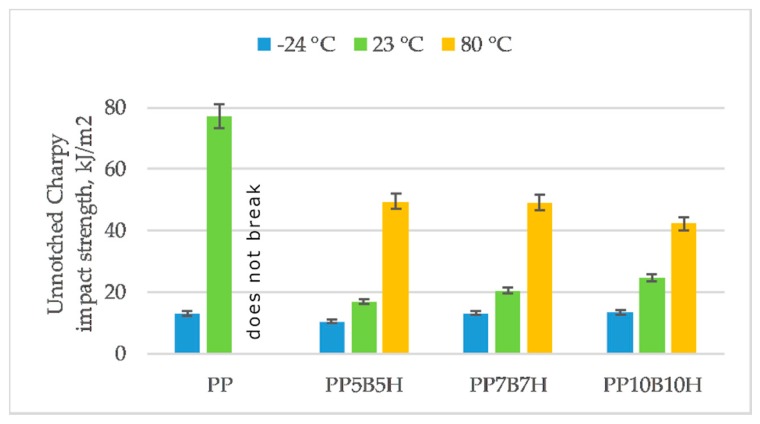
Un-notched Charpy impact strength of tested materials at −24, 23, and 80 °C.

PP has different properties at various temperatures, which limits its technical applications. Specialist literature data show that the glass transition temperature of homopolymer PP is −10 °C [21]. Below this temperature, the polymer becomes brittle and rigid. The influence of temperature on the mechanical properties change is presented in Figure 3, Figure 4, Figure 5 and Figure 6. Tensile strength, tensile modulus, strain at break, and un-notched Charpy impact strength were determined. Results obtained from the test carried out at ambient temperature showed that the tensile strength increased by 56%, and the tensile modulus increased by 74%, for composites with 10 wt% fillers. The highest tensile strength was observed for composites with 20 wt% fillers (54.2 MPa). An increase in the temperature caused PP to become more ductile; consequently, tensile strength and tensile modulus decreased at 80 °C. The addition of fillers caused a significant decrease in elongation; however, reinforced composites did not show significant differences in strain at break at various temperatures. The highest values were obtained for composites tested at −24 °C. Composites reinforced by lignocellulosic fillers were characterized by low strength properties and an increase of tensile modulus. Demirer et al. [22] studied the usability of ground HSs in PP matrix composites. The addition of ground HSs caused a decrease in tensile strength and an increase in Young’s modulus. Tensile strength for composites with 20 wt% HSs was 27.2 MPa, and tensile modulus was 1925 MPa. Composites reinforced with BFs had better mechanical properties. Tensile strength and tensile modulus was 70.7 MPa and 6114 MPa, respectively, for composites with 23 wt% BFs [23]. In our study, the addition of the two kinds of fillers caused an increase in both tensile strength and tensile modulus. However, mechanical properties increased primarily due to the addition of BFs.

The Charpy impact test measures the amount of energy absorbed by a composite during fracture. Neat PP is characterized by high impact strength. The addition of fillers caused a significant decrease of the impact strength; however, further addition of fillers up to 20 wt% affected an increase of impact strength. This increase of the impact strength is caused by the pull-out of fibers and the friction on the interphase. This was expected because HS is a relatively hard material and it could result in a decrease of the impact strength. The impact strength also depends on the pull-out of fibers, matrix fracture, and debonding of fibers and matrix. At lower temperatures, composites become more brittle and the impact toughness is low. At −24 °C, unfilled PP exhibited an 83% drop in impact strength. An addition of 10 wt% fillers caused a lower drop in impact strength at −24 °C, which was 37%. An increase in temperature caused that material to become more ductile; as a consequence, the impact toughness was higher. Unfiled PP at elevated temperatures did not break and composites with 10, 15, and 20 wt% fillers increased by 193%, 140%, and 42%, respectively.

### 3.3. Thermal Conductivity and Coefficient of Thermal Expansion

**Figure 7:**
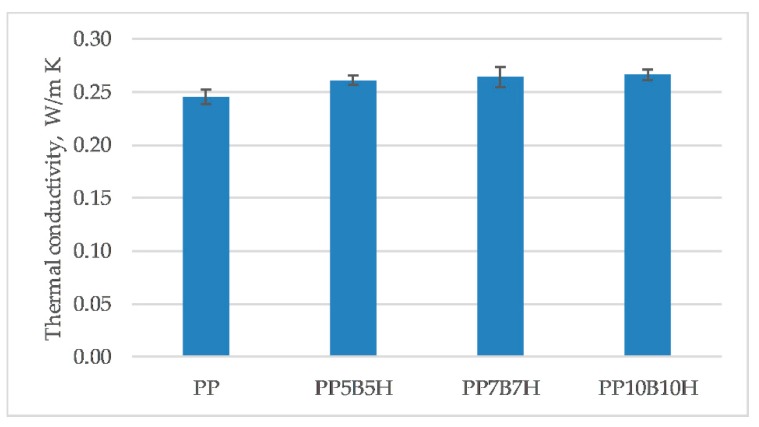
Thermal conductivity of the tested composites.

Figure 7 shows the thermal conductivity of the tested composites. Thermal conductivity is the capacity of materials to transfer heat from one point to another. PP is characterized by high resistance to electricity and can be classed as a good isolator. The addition of fibers caused an increase of thermal conductivity. The highest value was observed for composites with 20 wt% fillers, where thermal conductivity increased by 8%. Idicula et al. [24] studied the effect of fiber treatment on thermal conductivity. They examined whether sodium hydroxide treatment of fibers increased thermal conductivity. The addition of treated HSs could increase the thermal conductivity of hybrid composites.

**Figure 8:**
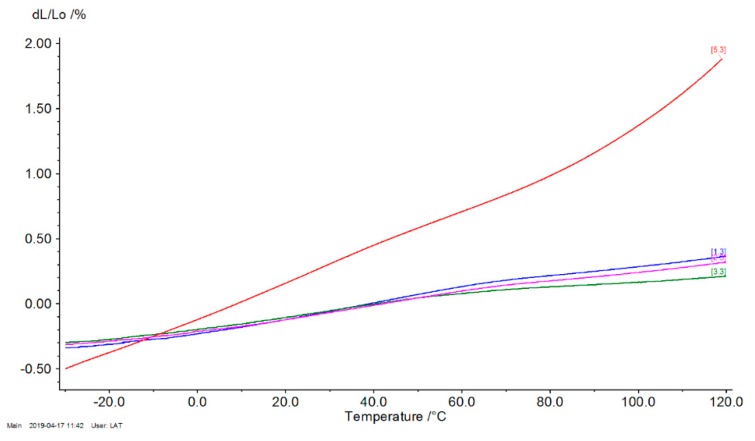
Thermal expansion curves of the tested composites: red line—polypropylene (PP), blue line—PPB5H, pink line—PP7B7H, and green line—PP10B10H.

Figure 8 presents thermal curves of the tested composite. Table 2 presents LCTE at temperatures from −20 to 50 °C. To achieve dimensional stability, a low LCTE is required. The linear coefficient decreased for all composites. It decreased by approximately 150% and 200% for PP5B5H and PP10B10H, respectively. The use of HS and BF helped to lower the overall LCTE values for hybrid composites.

### 3.4. Creep Test

**Figure 9:**
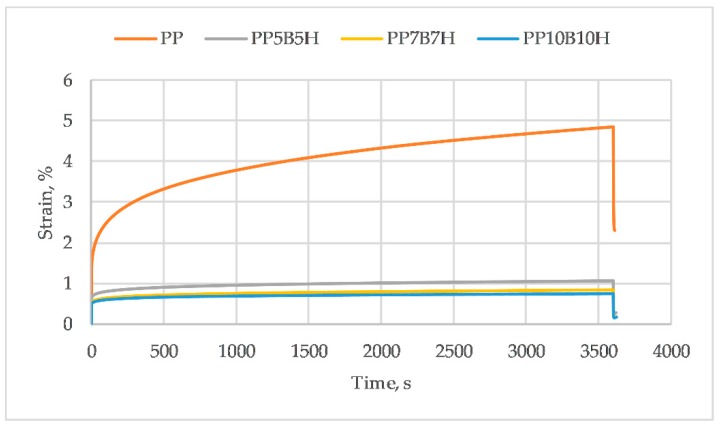
The effects of basalt fibers (BF) and hazelnut shell (HS) application level on the creep behavior of composites.

When composites are exposed to a long-lasting dead load, the creep behavior becomes a critical parameter. The creep affects the dimensional stability of composites. Figure 9 presents the effect of an addition of fillers on the creep behavior of composites. PP exhibits other creep-like thermoplastic materials. As the fillers content increased, the creep strain was reduced. This behavior showed a noticeable improvement in the creep stability of the hybrid composites that was induced by the presence of rigid BFs and HSs.

### 3.5. Fractographic Investigation

**Figure 10:**
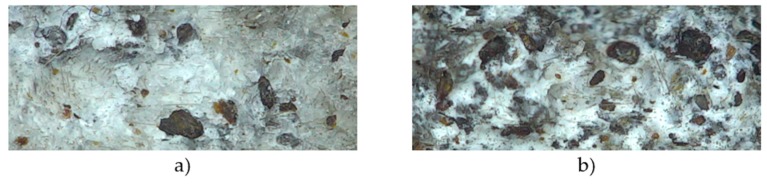
Optical micrographs of tensile fractured surfaces of (**a**) PP5B5H and (**b**) PP10B10H with 20× magnification.

The morphology of the fractured surfaces of tensile specimens was observed by the used SEM and optical microscope. In Figure 10, we can observe HS particles of different sizes. BFs are not clearly visible at this magnification. Figure 10a presents a composite with 5 wt% BFs and 5 wt% HSs. HS particles are not uniformly distributed, and, in this magnified fragment, less of them can be detected, in contrast to the composite with 10 wt% BFs and 10 wt% HSs.

**Figure 11:**
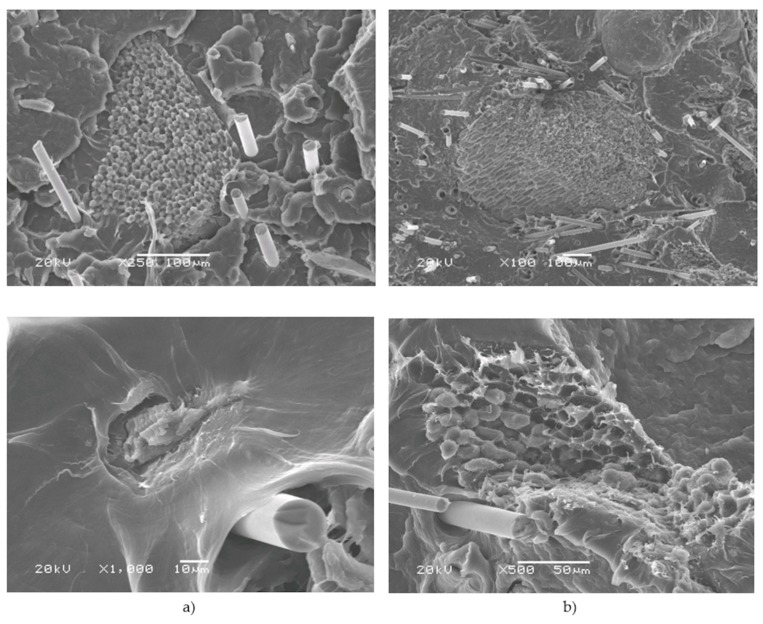
Scanning electron microscope (SEM) micrographs of tensile fractured surfaces of (**a**) PP5B5H and (**b**) PP10B10H.

From the observations of SEM images (Figure 11), it may be concluded that HS particles formed agglomerates in a polymer matrix, which contributes to the deterioration of mechanical properties. The dispersion of HS in PP can be improved by the addition of a promoter of mixing. Furthermore, HSs can be used in the form of granules. BFs were observed with the measured diameter of 17 μm. Hazelnut shell is a lignocellulosic material with an irregular surface. The adhesion between HS particles, BFs, and the matrix was strong; however, there were voids around the BFs and fiber pullouts are noticeable. HS particles are well-embedded in the polymer matrix. In Figure 11a, ductile fracture occurs. In Figure 11b, a larger amount of pulled fibers is noticeable. This resulted in increased impact strength and decreased mechanical properties.

### 3.6. The Influence of Water Absorption and Thermal Aging

**Figure 12:**
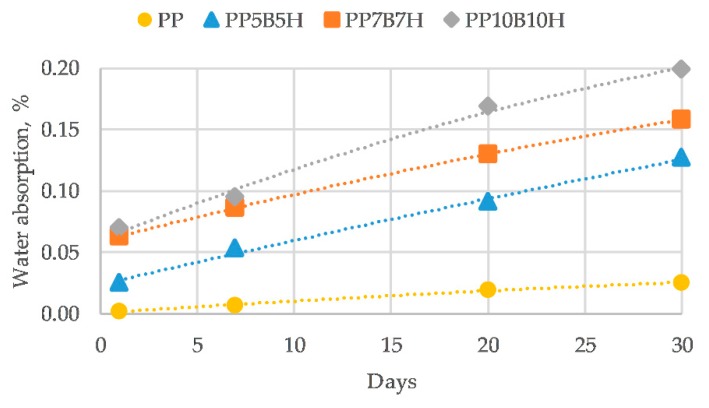
Water absorption of the tested materials.

Figure 12 presents the water absorption of the tested materials. Neat PP had a very low water absorption of about 0.03%. Water absorption increased with a growing filler content; however, the introduction of BFs in hybrid composites provided improved water absorption behavior. The increase of water absorption may be caused by an increased interfacial void between BF and the matrix, and because of the hydrophilic HS porous structure, which could accommodate water. This leads to a bad fiber/matrix interface, which results in a reduction in the mechanical properties of the composites. MAPP as a coupling agent is commonly used to improve fiber/matrix interface properties, and hence it reduces water absorption [25,26].

**Figure 13:**
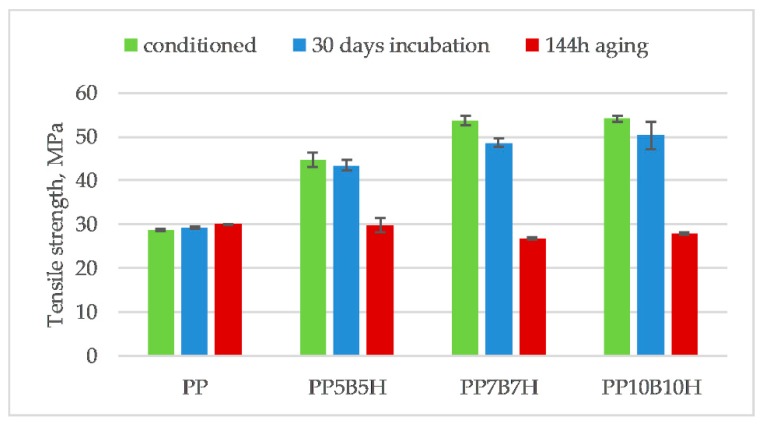
Comparison of tensile strength of PP-based composites before and after 30-day incubation in water and 144 h of aging.

**Figure 14:**
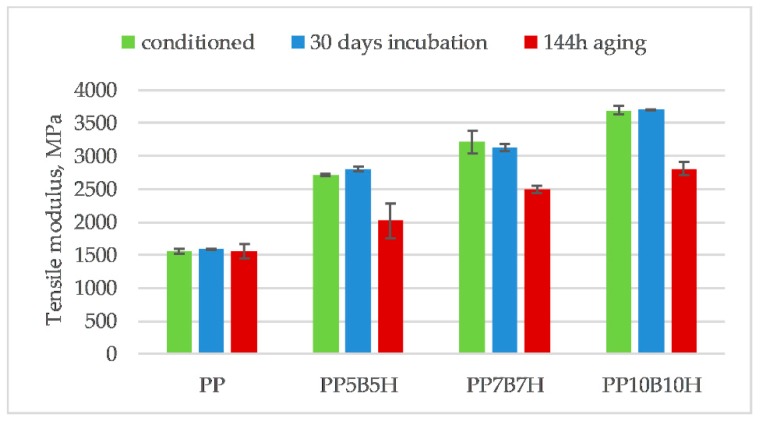
Comparison of tensile modulus of PP-based composites before and after 30-day incubation in water and 144 h of aging.

**Figure 15:**
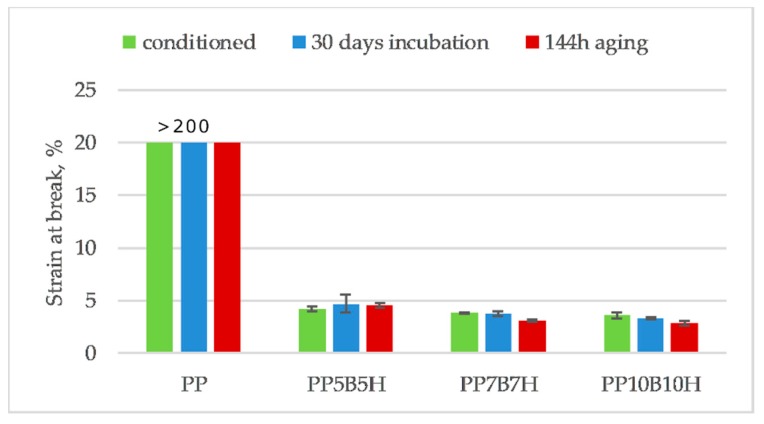
Comparison of strain at break of PP-based composites before and after 30-day incubation in water and 144 h of aging.

Figure 13, Figure 14 and Figure 15 compare the changes in tensile strength, tensile modulus, and strain at break, before and after they were subjected to the 30-day incubation in water, and after 144 h of aging. After incubation in water, the tensile strength decreased, which was expected due to the addition of lignocellulosic filler. Moreover, the water absorption would weaken the interfacial bonding between the fillers and polymer. Water absorption causes debonding of the interfacial adhesion between the filler and the matrix. After incubation in water, PP10B10H composites showed a reduction in tensile strength by approximately 7%. The effect of water absorption on tensile properties of hybrid composites was not significant. The tensile modulus increased for composites; it could be a result of swelling HS particles after the water absorption. Swelling could increase the contact area between the HS and the matrix.

A composite’s behavior during service conditions, analyzed by conducting aging, is one of the critical factors that affects its end use. The processes occurring in the materials during aging had an adverse effect on their strength properties. After aging, the tensile strength and tensile modulus decreased. There was no significant effect of aging on the tensile strength for unfilled PP. For composites with 10 and 20 wt% fillers, various physical and chemical factors can cause irreversible structural changes to occur in polymers, which cause a decrease in molecular weight (degradation) or a change in the chemical composition (destruction). Table 3 shows the weight changes before and after aging. The tested composites reduced weight, except for neat PP.

## 4. Conclusions

The main conclusion is that HSs can be used as a filler in hybrid PP composites. HS-reinforced composites are characterized by low strength properties. However, the addition of both BFs and HSs caused an increase in tensile strength and tensile modulus. The tensile strength increased by 56% and the modulus of elasticity increased by 74% for composites already reinforced by 10 wt% of fillers. The results showed that addition of fillers successfully improved the thermal and creep stability. The effect of water absorption on mechanical properties of composites was not significant. After thermal aging, the tensile strength and modulus of elasticity significantly decreased. SEM micrographs showed the morphology of HSs and BFs. Debonding and pull-out of fibers, noticed for composites with 20 wt% of fillers, caused a decrease in strength properties and an increase in impact strength. To improve the adhesion between the BFs and the matrix, further studies are planned.

## Figures and Tables

**Table 1:** Composition and basic properties of tested composites.

Symbol	Composition	Density, g/cm^3^	Vicat Softening Temperature, °C
**PP**	100% polypropylene HP 500N	0.886	150.6
**PP5B5H**	87 wt% PP + 5 wt% BF + 5 wt% HS + 3 wt% MAPP	0.992	158.0
**PP7B7H**	82 wt% PP + 7.5 wt% BF + 7.5 wt% HS + 3 wt% MAPP	0.993	161.2
**PP10B10H**	77 wt% PP + 10 wt% BS + 10 wt% HS + 3 wt% MAPP	0.997	161.8

**Table 2:** Linear coefficient of thermal expansion of tested materials.

Specimen	Linear Coefficient of Thermal Expansion (−20 to 50 °C) α × 10^−6^, 1/K
PP	136.6
PP5B5H	54.8
PP7B7H	47.4
PP10B10H	45.8

**Table 3:** The weight changes of the tested composites before and after aging.

	PP	PP5B5H	PP7B7H	PP10B10H
	g			
Before drying	36.78	32.9	34.13	34.91
After drying at 70 °C	36.79	32.84	33.96	34.71
Percentage weight difference	−0.03%	0.18%	0.50%	0.57%
